# The effect of the proportion of Modern European ancestry on grower and sow performance of pigs in smallholder systems in Uganda

**DOI:** 10.3389/fgene.2023.1123826

**Published:** 2023-09-25

**Authors:** Brian Martin Babigumira, Johann Sölkner, Gábor Mészáros, Maria Wurzinger, Christina Pfeiffer, Craig R. G. Lewis, Ben Lukuyu, Emily Ouma, Karen Marshall

**Affiliations:** ^1^ Department of Sustainable Agricultural Systems, Division of Livestock Sciences, University of Natural Resources and Life Sciences, Vienna, Austria; ^2^ International Livestock Research Institute, Kampala, Uganda; ^3^ PIG Austria GmbH, Steinhaus, Austria; ^4^ Pig Improvement Company, Barcelona, Spain; ^5^ International Livestock Research Institute, Nairobi, Kenya

**Keywords:** pig, growth, litter size, genotype, smallholder, Uganda

## Abstract

Several factors, including breed, lead to divergent performance of pigs for production and reproduction traits in different environments. A recent genomics study showed that Modern European (ME) pig breeds contribute to the ancestry of smallholder pigs in the Hoima and Kamuli districts, Uganda. These pigs were also involved in a longitudinal study with several traits recorded, including 540 body weights (WT) of 374 growing pigs, 195 records of total number of piglets born alive (TBA) of 157 sows, and 110 total number weaned (TNW) records of 94 sows. Linear mixed-effects models were used to test for the significance of environmental effects, including housing system, geographic location, and the season when the events occurred as well as animal-specific effects like age, sex, parity, and farrow-to-weaning interval. Stepwise model reduction starting from models with all main effects and pairwise interactions was applied. The final models were then expanded to include proportions of Modern European (ME) ancestry for the subset of animals genotyped, following genomic ancestry analysis based on a Porcine 50K SNP Chip. ME ancestry proportions ranged from 0.02 to 0.50 and were categorized into three classes (low/medium/high ME) based on 33.3% quantiles. The effects of ME classes on WT and TBA were not significant. ME showed a significant effect on TNW. Sows with a high proportion of ME weaned 2.4 piglets more than the low group, the medium ME group being intermediate. This study used genomic data to investigate the effects of genetic ancestry on the performance of smallholder pigs in Uganda. The proportion of Modern European ancestry did not exceed 0.50, therefore not allowing for the comparison of local versus pure “exotic” types of pigs. For the range of ancestries observed, which is the relevant one for current smallholder systems in Uganda, differences were small for the body weight of growing pigs and the number of piglets born alive, while higher proportions of ME ancestry resulted in significantly more piglets weaned. The availability of genotypes of a higher number of growing pigs would have been beneficial for drawing conclusions on the effect of ME ancestry on the growth rates of smallholder pigs in Uganda.

## 1 Introduction

Pork is an important source of animal protein and represents 30%–40% of the meat consumed globally (FAO, 2014). The top pork-producing countries in Africa include Nigeria, Malawi, and Uganda (FAOSTAT, 2021). The national herd of Uganda is estimated at 4.2 million pigs (UBOS, 2020), and the *per capita* consumption of pork is 3.4 Kg (FAOSTAT, 2018). Smallholder farmers represent the majority of pig producers and pigs are kept for savings/insurance and income (Babigumira et al., 2019). Small herds of variable size are kept from which piglets, slaughter animals, or both are produced (Ouma et al., 2015; Ouma, 2017). Pig breeding is unstructured, and services like artificial insemination are not commonly used. Most farmers rely on the services of a village boar for a fee to breed their sow (Dione et al., 2014). Performance traits related to reproduction (litter size), growth, and disease resistance are important to smallholder farmers (Babigumira et al., 2019). All these constraints have implications on the performance of pigs in these typically low-input smallholder systems.

Previous studies on the performance of pigs in Africa have been done under differing production conditions and have, to a great extent, relied on pig breed composition as reported by farmers or research stations, that is, local, crossbred, and exotic (Adebambo and Dettmers, 1982; Affentranger et al., 1996; Ajala, 2007; Kagira et al., 2010; Muhanguzi et al., 2012; Okello, 2015; Dotche et al., 2020a). However, there is consensus that local pigs in Africa were introduced and are of European and Asian ancestries (Blench, 2000; Ramirez et al., 2009; Noce et al., 2015; Dotche et al., 2020a; Babigumira et al., 2021). Additionally, it becomes difficult, missing pedigree information withstanding, to account for genetic effects on an animal’s performance, more so in admixed populations. Nevertheless, advances in bioinformatics and sequencing technologies have made it possible to overcome such hurdles. To the best of our knowledge, the study by Babigumira et al. (2021) is the first in Uganda to both decipher and quantify the ancestry of smallholder pigs using SNP Chip data (Babigumira et al., 2021). Babigumira et al. (2021) analyzed the ancestries of pigs kept by smallholder households in Uganda with Old British, Modern European, Iberian, Duroc, and Chinese pigs as potential ancestral populations and found that the pigs were mostly a mix of Old British and Modern European (ME) types. The current study is a follow-up to the study by Babigumira et al. (2021). Both studies were conducted as part of a longitudinal survey of smallholder pig herds in the districts of Hoima and Kamuli, Uganda, under a larger project. Here, we incorporated genomic information and statistically tested the effects of ME ancestry (ranging from 2%–50%) on phenotypes recorded on these smallholder pigs in Kamuli and Hoima districts, Uganda. Our results highlight the role of the environment in the performance of pigs in smallholder herds and imply a holistic approach when intervening in smallholder pig production.

## 2 Materials and methods

### 2.1 Study sites and households

The study sites selected were Hoima and Kamuli districts due to the importance of pig-keeping to smallholder’s livelihoods in these districts. Household selection proceeded as follows. For selected sub-counties within Hoima and Kamuli districts, a full list of pig-keeping households was obtained in collaboration with the district extension staff. From here, 300 households were randomly selected and surveyed for key information on their household pig enterprise type, including the main breed type of pig kept (local, cross-bred of local and exotic, and exotic) and type of pig housing (free-range and tethered versus housed). Households’ pig enterprises were then classified based on combinations of main breed-type kept and housing practiced (as local-tethered, cross-breed-tethered, exotic-tethered, cross-bred-housed, and exotic-housed) with the final set of 200 project households purposively selected from these groups, such that each enterprise type had approximately an equal number of households. The 200 households were in 30 villages in 26 parishes across 8 sub-counties in the 2 districts.

### 2.2 Ethics statement

This research was approved by the Uganda National Council of Science and Technology (UNCST), the Research Ethics Committee of the Vector Division of the Ministry of Health (VCD-REC), Uganda, the Research Ethics Committee (IREC), and the Institute Animal Care and Use Committee (IACUC) of the International Livestock Research Institute (ILRI). Farmers’ participation in the study was voluntary.

### 2.3 Genotypes

The breed composition (genotypes) of the pigs used in the current study had been inferred by admixture analysis in a related study (Babigumira et al., 2021). Briefly, the genotyping process in Babigumira et al. (2021) proceeded as follows. Hair samples were taken from a random sample of pigs kept by 148 of the 200 smallholder households in the districts of Hoima and Kamuli. Further, pigs phenotypically representative of “local” pigs were also sampled from smallholder households in three other districts, namely, Soroti, Kumi, and Paliisa. Genotyping was done using the Geneseek Genomic Profiler Porcine 50k SNP chip and ancestry proportions were inferred by admixture analysis using ADMIXTURE 1.3 (Alexander et al., 2009). The pigs were found to have a mix of Old British and Modern European (ME) ancestries. Large White and Landrace pig breeds contributed to most of the ME ancestry proportions which were between 0.02 and 0.5 (Babigumira et al., 2021).

### 2.4 Data collection

Data were collected on all pigs present within the project household at the time of the survey visit. Initially, a pig census survey was performed (October to November 2018) with all pigs within the households tagged and demographic data on each pig obtained (including age, sex, and breed, and for sows their parity, as per farmer recall) using a structured survey. From here the household pig herds were longitudinally monitored (December 2018 to March 2020). During the longitudinal monitoring, the households were visited eight times at intervals between 1 and 3 months depending on the weather and related field activities, and information on their household pig enterprises and pigs was recalled to the previous visit, using a structured survey. Data captured during the longitudinal monitoring included (amongst others) farrowing and weaning events, health, nutrition (feeds and feeding practices), herd dynamics (entries and exits), pig transactions (sales and purchases), housing systems, and morphometric and body weight measurements.

This study focused on an analysis of growth and fertility traits [total number of piglets born alive (TBA) and total number of piglets weaned (TNW)]. Body weight (WT) measurements were taken at birth, when possible; otherwise, the birth date was recalled by the farmer and the weight of the pig was measured during the visit. Pigs were weighed every subsequent visit until the animal exited the farm (through sale or death) or until the end of the survey. The WT was measured using a digital weighing scale (Brand: Crane, range of measurement: 1–200 Kg and accuracy: 0.12 kg). Heart girth (HG) and body length (BL) measurements were taken at the time of weighing each pig. Sow fertility data collected included farrowing and weaning dates and litter sizes at birth (TBA) and weaning (TNW). The data was entered into the Census and Survey Processing System (CSPro) (U.S. Census Bureau, 2019) and reposited in a SQL database on the ILRI data portal (Rutto et al., 2019).

### 2.5 Data analysis

We analyzed the influence of a range of effects (described below) on variation in growth and litter size of pigs. All effects and their possible pairwise interactions were tested at a significance level of 0.05 by a linear mixed effects model using the lme4 package in the R environment (Bates et al., 2014; R Core Team, 2020). Results from the lme4 package were visualized using the lmerTest R package (Kuznetsova et al., 2017). Further, to account for population structure, we generated a genomic relationship matrix and included it in the mixed model analysis using the R package lme4qtl (Ziyatdinov et al., 2018). Least-squares means (LSM) were estimated and compared pairwise by the Kenward-Roger method and Tukey *p*-value adjustment method for comparing multiple estimates using the lsmeans R package (Lenth, 2016).

#### 2.5.1 Description of variables

Body weight (WT) and litter size at farrowing (TBA) and weaning (TNW) were continuous dependent variables. The independent variables of interest were the housing system, geographic location of the farm, season, sex (for growers), farrow-to-weaning interval, and parity (for sows). The pigs in each household were managed under one of three housing systems: free-range (only for growers), tethered, and housed. The proportion of Modern European (ME) was inferred in a previous study (Babigumira et al., 2021) and was categorized into low, medium, and high classes based on 33.3% quantiles. The season was defined as dry or wet based on the seasons of Uganda to which the month of farrowing or weaning (for sows) or weighing (for growers) belonged. Uganda majorly has two wet seasons: March to May and September to December (Caffrey et al., 2013; Mubiru et al., 2018). Parity was defined as “1” for a primiparous and “2+” for a multiparous sow. The farrow-to-weaning interval was a continuous variable computed in days and then categorized based on 33.3% quantiles. Age was a continuous variable while sex was a categorical variable (female or male). Genotypes were available on only 11.0% of growing pigs with body weights (43 of 374) due to the inability to hair sample very young pigs and their absence at the next survey visit (e.g., due to sale or death). In contrast, 66% (103 of 157) of the sows were genotyped. The 43 genotyped growing animals with 94 records on WT were assigned to three ME classes on 33.3% quantiles (low ≤ 0.181, 0.181 > medium < 0.28, and high ≥ 0.28). The sows were assigned to three ME classes based on 33.3% quantiles (low ≤ 0.153, 0.153 > medium < 0.289, and high ≥ 0.289). The number of animals in each category of the variables is presented in Table 1.

**TABLE 1:** Number of animals in each category of environmental and genetic effects.

Characteristic	Levels	Sows (N)		Growers (N)
		Farrow	Wean	
Geographic location	Kamuli	91	61	319
	Hoima	66	34	55
Season	Dry	59	32	226
	Wet	107	67	254
Housing system	Housed	43	26	110
	Tethered	109	69	70
	Free-range	0	0	131
Parity	1	98	58	NA
	2+	77	43	NA
Sex	Male	NA	NA	172
	Female	157	95	191
ME	Genotyped	103	67	43
ME classes	Low	37	21	13
	Medium	34	24	13
	High	32	22	17
Farrow-to-weaning interval	Low	NA	35	NA
	Medium	NA	35	NA
	High	NA	38	NA

#### 2.5.2 Statistical models

A range of effects potentially affecting the traits under study, including geographical location, housing system, and season, was included in the linear mixed effects statistical models employed. As only part of the animals with phenotypes were also genotyped for the prediction of levels of ME ancestry, the following strategy of analysis was employed.

First, mixed linear models with fixed environmental effects and all their pairwise interactions as well as the random effect of animals, accounting for repeated measurements, were tested. A stepwise procedure for model reduction was followed, excluding non-significant interaction terms one by one and then excluding non-significant main effects not involved in any of the interactions. The model reduction was based on Pearson’s chi-square (ꭓ2) statistic with a threshold of 0.05.

Second, the resulting model was then employed adding the proportion of Modern European ancestry (ME: low, medium, and high) as well as its pairwise interactions with the other fixed effects in the final environmental effects model. Non-significant pairwise interaction terms of these environmental effects and ME were also excluded in a stepwise manner to arrive at the final model. Therefore, the results for the fixed environmental effects presented here are derived from the initial dataset with more observations while the effects of ME ancestry and its interactions come from the smaller dataset of genotyped animals (Ziyatdinov et al., 2018). We run the final models fitting ME as a categorical variable and a continuous variable.

##### 2.5.2.1 Grower performance

A total of 540 WT records from 374 animals with indicators of age, geographic location, sex, pig housing system, and season were available. The number of animals with one, two, three, and four records was 252, 83, 34, and 5. For the 374 animals, the ranges of WT, HG, BL, and age were 0.7–49.0 Kg, 5.0–73.0 cm, 14.0–91.0 cm, and 7.0–210 days, respectively. The correlations between WT and the two morphometric measurements (HG and BL) ranged from 0.74 to 0.92 (Table 2).

**TABLE 2:** Correlation between WT, HG, and BL.

	WT (Kg)	HG (cm)	BL (cm)
WT (Kg)	1.00		
HG (cm)	0.74	1.00	
BL (cm)	0.75	0.92	1.00

The significance of the environmental effects on WT and all pairwise interactions were investigated using model (Eq. 1).

$$WT_{ijklmn}=A_{i}+G_{j}+S_{k}+H_{l}+W_{m}+AN_{n}+all\;pairwise\;interactions+\in_{ijklmn}$$ (1)

Where $WT_{ijklmn}$ is the body weight of the $n^{th}$ animal; $A_{i}$ is the $i^{th}$ age in days (covariate); $G_{j}$ is the $j^{th}$ geographical location; $S_{k}$ is the $k^{th}$ sex; $H_{l}$ is the $l^{th}$ pig housing system; $W_{m}$ is the $m^{th}$ season in which the animal’s body weight was measured; $AN_{n}$ is $n^{th}$ grower (random effect); $\in_{ijklmn}$ random residual effect.

##### 2.5.2.2 Sow performance

The effect of season, geographic location of the farm, pig housing system, and parity as fixed effects and the sow as a random effect on the total number of piglets born (TBA) which is 195 observations from 157 sows, and on the total number of piglets weaned (TNW) which is 110 observations from 94 sows was investigated using model (Eq. 2).

$$TBA_{ijkln},TNW_{ijklmn}=S_{i}+G_{j}+H_{k}+P_{l}+I_{m}+AN_{n}+all\;pairwise\;interactions+\in_{ijkln},\in_{ijklmn}$$ (2)

Where $TBA_{ijkln}$ is the total number of piglets born alive and $TNW_{ijklmn}$ is the total number of piglets weaned; $S_{i}$ is the $i^{th}$ farrowing or weaning season; $G_{j}$ is the $j^{th}$ geographic location of the farm; $H_{k}$ is the $k^{th}$ housing system; $P_{l}$ is the $l^{th}$ parity; $I_{m}$ is the $m^{th}$ farrow-to-weaning interval; $AN_{n}$ is the $n^{th}$ sow (random effect); $\in_{ijklmn}$ is a random residual effect.

## 3 Results and discussion

### 3.1 Description of body weight and litter size

**FIGURE 1:**
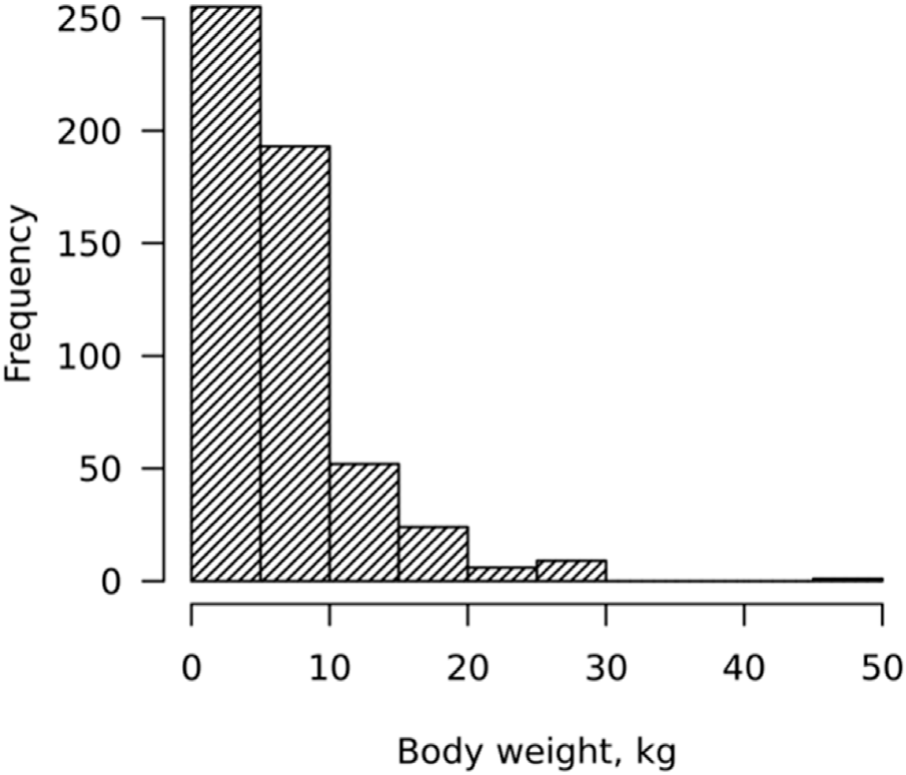
Distribution of body weight (WT).

Most growing animals (92.5%) weighed less than 10 Kg (for HG, BL, and age, the weights were less than 68 cm, 79 cm, and 200 days, respectively) due to heavier animals being sold from the household prior to the time of visits (Figure 1).

Note that the WT of eight animals with missing WT measurements but available HG and BL measurements were predicted using a multiple linear regression equation based on (Eq. 3).

WT=−9.45091+0.40756×HG+0.02152×BL (3)

HG and BL explained 61% of the variation of WT (R-squared = 0.61)

**FIGURE 2:**
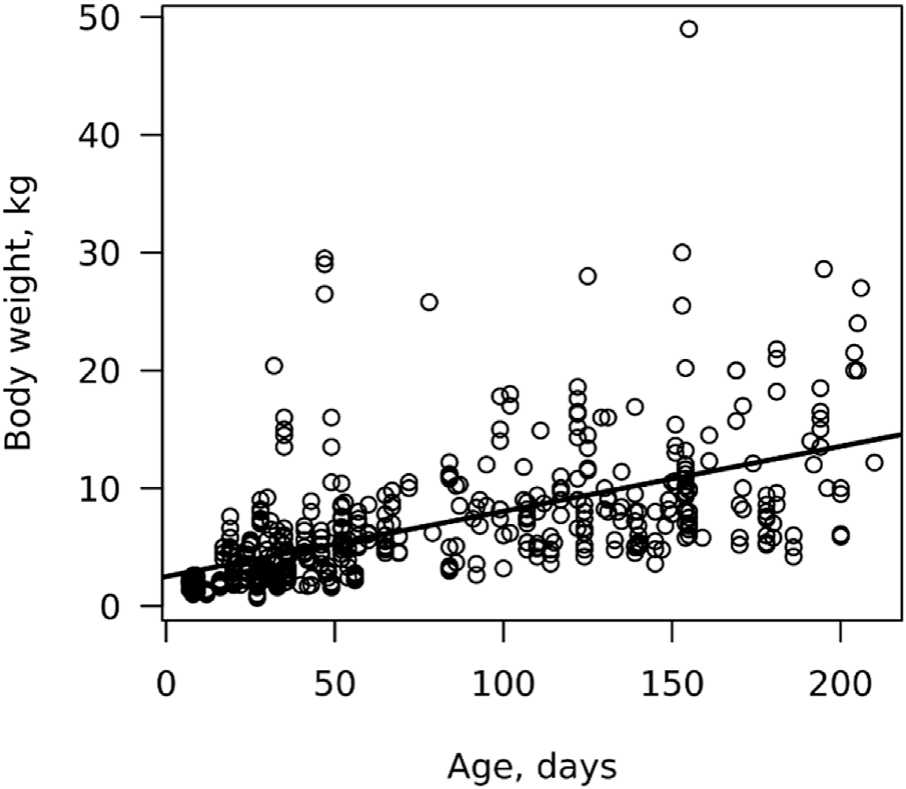
Weight-for-age of growing pigs.

The relationship between body weight and age is shown in Figure 2. The WT was very variable with age with some animals at either end of the spectrum. Variability in WT of growing pigs has also been reported in the Philippines (More et al., 1999) and Kenya (Mutua et al., 2011), as well as in commercial herds (López-Vergé et al., 2018).

**FIGURE 3:**
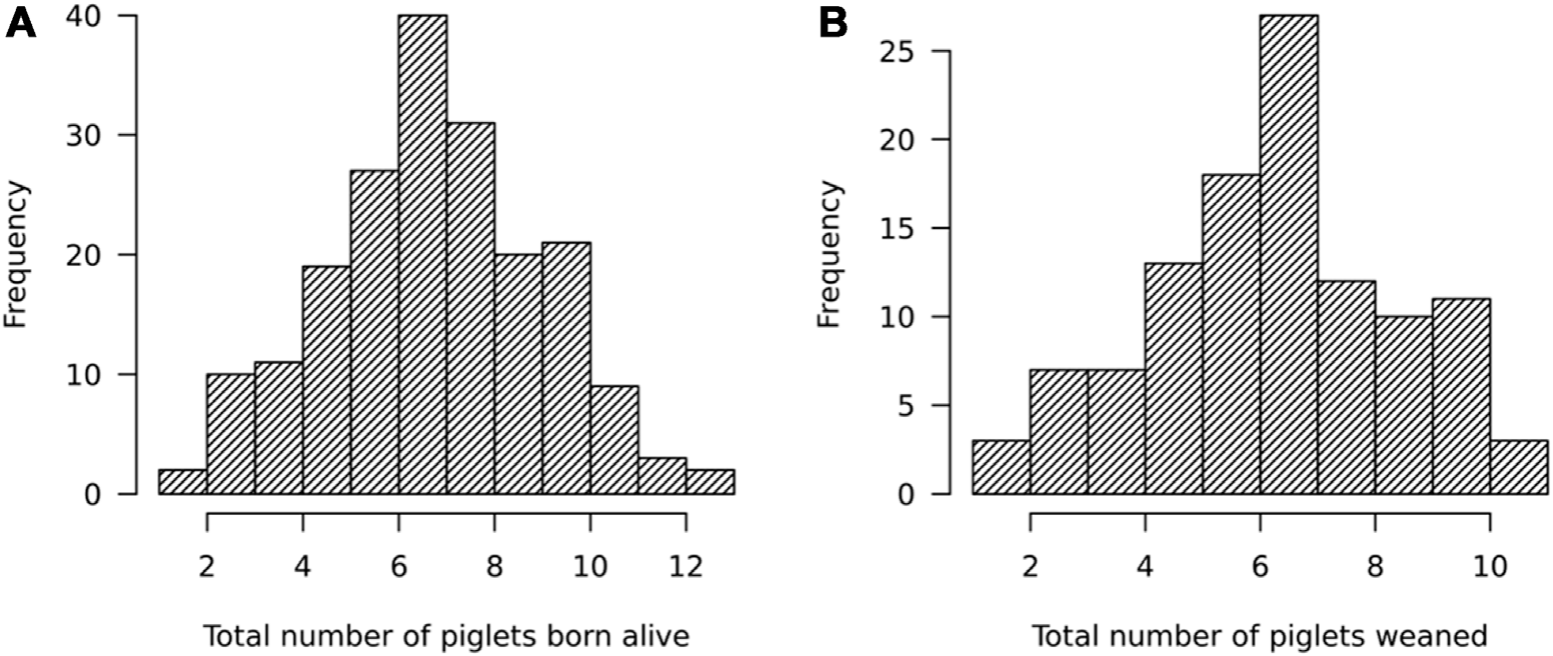
Distribution of **(A)** total number of piglets born alive (TBA) and **(B)** total number of piglets weaned (TNW).

For sows, a total of 195 litters with a mean ± standard deviation of 7.2 ± 2.3 (with a range from 1 to 13) had been farrowed by 157 sows between July 2018 and March 2020. The TBA values are comparable to those reported in India and Nigeria (Kumaresan et al., 2007; Abah et al., 2019) but lower than those reported in commercial herds in Uganda (Okello, 2015). A total of 110 litters of 94 sows had weaning records on the total number of piglets weaned, the season of farrowing, parity, geographic location of the farm, and the pig housing system practiced on the farm. The average size of weaned litters was 6.1 ± 2.2 (with a range from 1 to 11) piglets. The TNW values reported here are lower than those reported by Okello (2015). The litters were weaned between October 2018 and March 2020. The distribution of TBA and TNW is shown in Figure 3.

### 3.2 Models including environmental effects

#### 3.2.1 Grower performance

The final (reduced) model for growth performance contained the main effects and interaction terms presented in Table 3.

**TABLE 3:** Significance of effects and interaction terms retained in the reduced model for WT.

	χ2	DF	*p*-value
Age	196.095	1	<0.0001
Housing system	9.583	2	0.00830
Season	2.416	1	0.12011
Geographic location	0.629	1	0.42771
Age: season	17.751	1	0.00003
Age: geographic location	5.162	1	0.02308

The variances of the random effects, namely, animal and residuals were 7.762 and 11.521, respectively, translating to a repeatability of 0.67 of the body weight measurements. The average daily gain (ADG) derived from linear regression of weight on age was 55.2 g/day. The least-square means for WT by housing system are presented in Table 4. Pairwise comparisons showed significant differences between housing systems (free-range vs housed).

**TABLE 4:** The least-square means for WT by housing system.

Housing system	LS mean	SE
Free-range	6.31^a^	0.52
Tethered	7.36^a,b^	0.50
Housed	8.11^a^	0.42

^(a, b)^ LS means with different superscripts are significantly different.

The housing system had a significant effect on WT, and this could be attributed to the intensified management of housed pigs. Pigs in Tanzania were found to gain between 68 g/d when left to free-range, and 72 g/day when confined/housed (Lipendele et al., 2015). The ADG reported in our study is close to those reported in Benin (Kouthinhouin et al., 2009) but lower than the 77 g/day that was reported for smallholder pigs elsewhere in Uganda (Lule and Lukuyu, 2017). Furthermore, the ADG found in our study was much lower than those reported for pigs in Kenya (Mutua et al., 2011; Carter et al., 2013), Ghana (Darfour-Oduro et al., 2009), Zimbabwe (Chimonyo et al., 2010), and India (Kumaresan et al., 2007); the latter was mostly derived from feeding trials. Smallholder pigs are fed energy-rich but protein-deficient crop residues comprising root tubers and their vines or leaves, e.g., sweet potato and cassava (Carter et al., 2015). Feed shortages and poor-quality forages in the tropics contribute to slower pig growth (Mutua et al., 2012; Mutua et al., 2012; Levy, 2014; Levy, 2014). Age (Carter et al., 2013) was found to have a significant effect on WT as reported in our study.

#### 3.2.2 Sow performance

##### 3.2.2.1 Total number of piglets born alive

For TBA, the only significant effect retained was parity (χ2 = 5.8916; *p* = 0.01521). The variance components for the random effects, namely, animal and residual were 0.728 and 4.294, respectively, translating to a repeatability of 0.17. The least-square means for TBA by parity are shown in Table 5. Pairwise comparisons showed significant differences between classes of parity (*p* = 0.0173).

**TABLE 5:** The least-square means of TBA by parity.

Parity	LS mean	SE
1	6.85^a^	0.23
2+	7.62^b^	0.24

^(a, b)^ LS means with different superscripts are significantly different.

Multiparous sows farrowed 0.77 piglets more than their primiparous cohort. Litter size increased with each parity till around the fourth (Dotche et al., 2020b).

##### 3.2.2.2 Total number of piglets weaned

The significant fixed effects and interaction terms were retained stepwise (Table 6) from model (Eq. 3). The significant main effects and interaction terms are presented in Table 6.

**TABLE 6:** Significance of fixed effects and their pairwise interaction terms on TNW.

Effect	χ2	DF	*p*-value
Season	0.011	1	0.9166
Geographic location	3.486	1	0.0619
Housing system	5.584	1	0.0181
Parity	6.742	1	0.0094
Season: Geographic location	7.255	1	0.0071
Season: Parity	5.157	1	0.0232

Sows that farrowed in the wet season weaned 0.54 piglets less. The wet season rather than cold weather is associated with piglet mortality (Chiduwa et al., 2008). Multiparous sows weaned 1.6 piglets (*p* = 0.0013) more and this is attributed to the improvement in the mothering ability of the sow. The least-square means of TNW by geographic location, housing system, and parity are presented in Table 7. Pairwise comparison showed significant differences between different levels of each variable.

**TABLE 7:** The least-square means for TNW by geographic location, housing system, and parity.

Geographic location	LS mean	SE
Hoima	7.80^a^	0.41
Kamuli	6.57^b^	0.31
Housing system		
Housed	7.74^a^	0.43
Tethered	6.63^b^	0.28
Parity		
1	6.39^a^	0.29
2+	7.98^b^	0.42

^(a, b)^ LS means with different superscripts are significantly different.

### 3.3 Testing effects of the proportion of Modern European (ME) ancestry

We tested the effects of ME on only the genotyped animals with the GRM (using the lme4qtl package) and without the GRM (using the lme4 package). Given that we obtained the same results in either case, here, we report the results obtained using the lme4 R package (Bates, 2010).

#### 3.3.1 Grower performance

**FIGURE 4:**
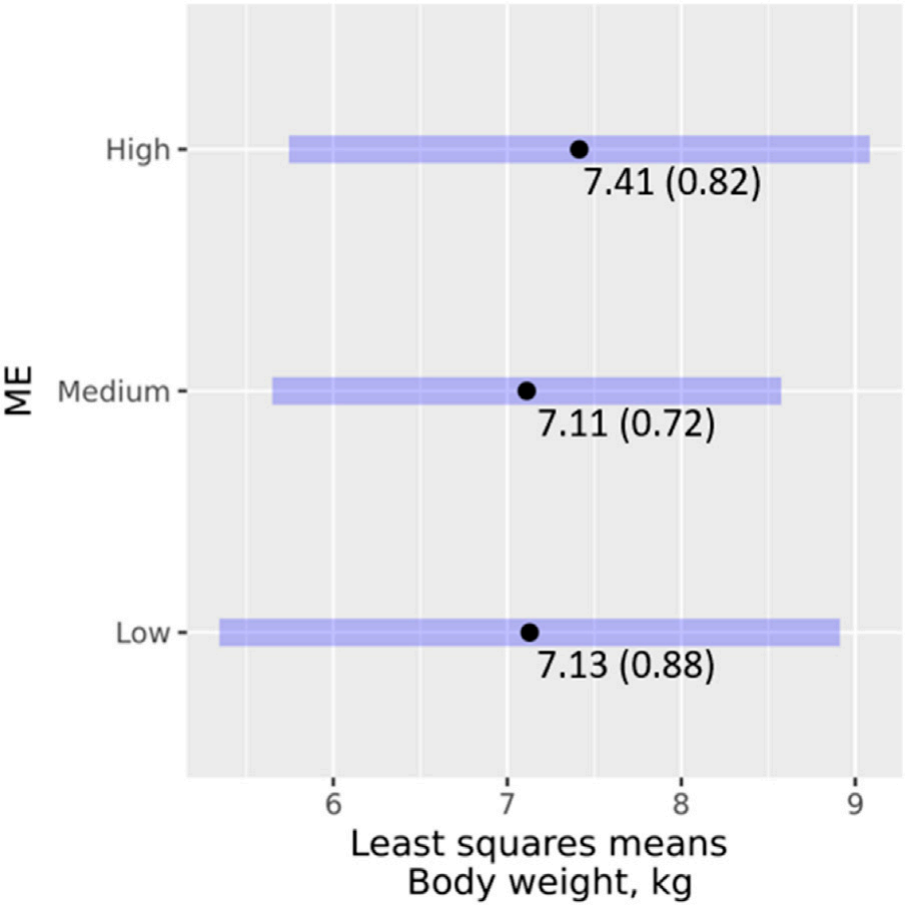
Effects of ME classes on WT, least square means (standard error), and their 95% confidence intervals.

A total of 94 WT records from 43 genotyped growing animals were available. The analysis of the effect of ME classes on WT showed that ME did not have a significant effect on WT (χ2 = 0.104, *p* = 0.949), and none of the pairwise interaction terms of ME with the other main effects was significant (*p* = 0.083 or higher). Figure 4 shows the least-square means and 95% confidence intervals of ME classes. Pairwise comparisons revealed non-significant (*p* < 0.05) differences between the ME classes. Further analysis with ME as a regressor also revealed neither it (χ2 = 0.001, *p* = 0.973) nor its interactions with the other effects (*p* = 0.489 or higher) in the model had a significant effect on WT.

It is generally accepted that exotic pigs weigh heavier than their indigenous counterparts. However, we found no significant differences in the effects of ME class on WT. It is likely that ME effects are confounded by other effects such as the housing system. Pig sties are usually provided by farmers capable of intensifying production, for example, by using improved breeds and providing better management (Dione et al., 2014; Ouma et al., 2015). This may partly explain the trend in body weight across the ME classes.

#### 3.3.2 Sow performance

##### 3.3.2.1 Total number of piglets born alive (TBA)

**FIGURE 5:**
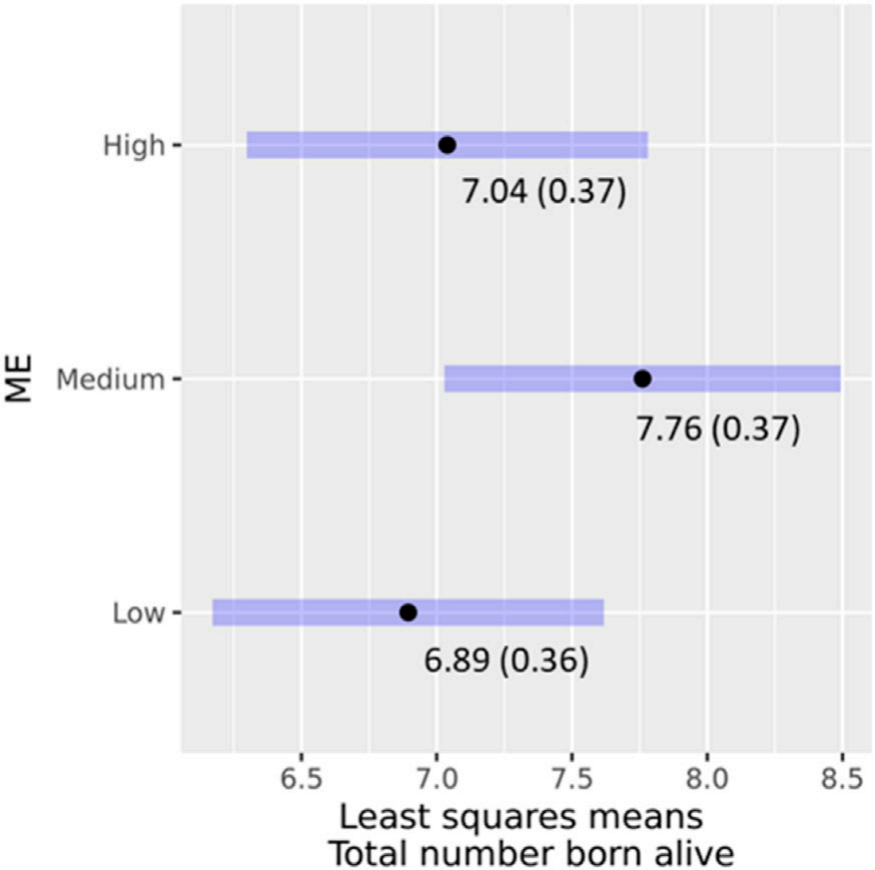
Effects of ME classes on TBA, least square means (standard error), and their 95% confidence intervals.

As only parity was significant after the reduction of model (Eq. 3) with the full phenotype data, the proportion of Modern European and its interaction term with parity was added for the analysis of data of genotyped animals. A total of 135 farrowing records that belonged to 103 genotyped sows were available for analysis. ME (χ2 = 3.2163; *p* = 0.20026) nor its interaction with parity (χ2 = 0.64804; *p* = 0.64804) had significant effects on TBA. The least-square means of ME and their 95% confidence intervals for TBA are presented in Figure 5. Sows in the ME medium and high groups farrowed 0.86 and 0.14 piglets more than those in the low group. Pairwise comparisons were significant between low and medium ME classes. A study in Cameroon that compared primiparous local versus exotic sows, e.g., Large White, reported lower litter size for the local sows though the breed effects were non-significant. However, the breed had a significant effect on the litter size of multiparous sows (Kouamo et al., 2015).

##### 3.3.2.2 Total number of piglets weaned (TNW)

**FIGURE 6:**
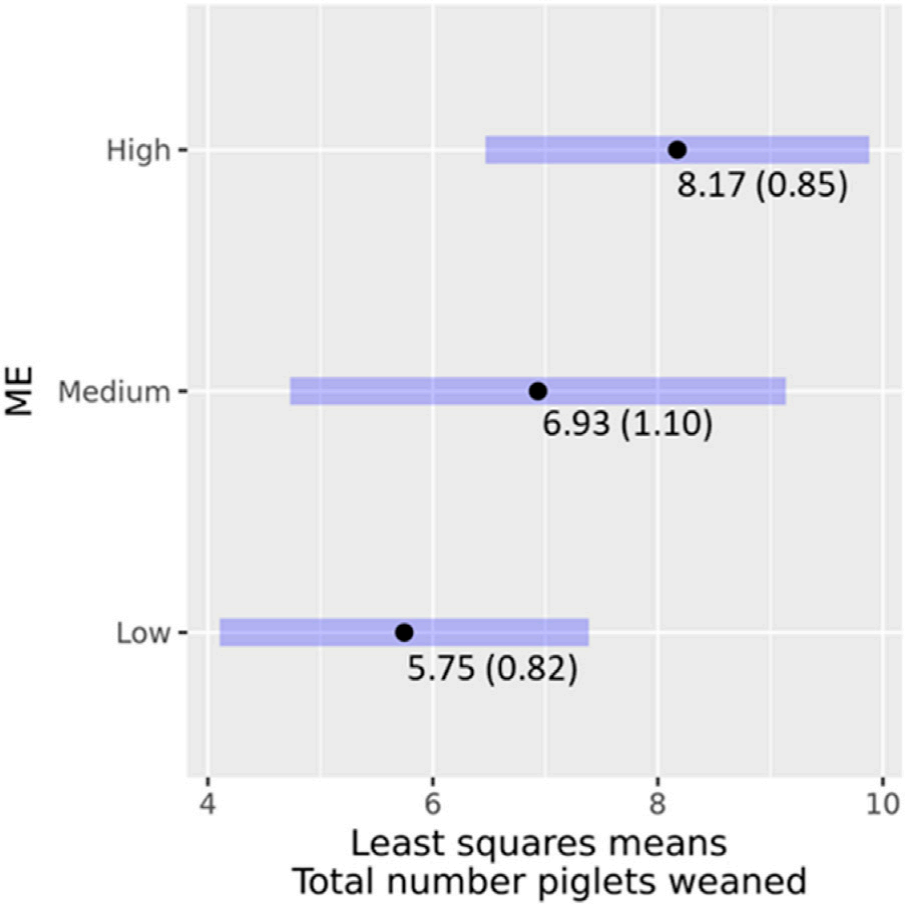
Effects of ME classes on TNW, least square means (standard error), and their 95% confidence intervals.

For TNW, a total of 80 weaning records that belonged to 67 sows were available for analysis. ME had a significant effect (χ2 = 10.3928; *p* = 0.005537) on TNW as were the interactions between ME and geographic location (χ2 = 6.8424; *p* = 0.032673). The LSMs for TNW by the interaction between ME and geographic location are shown in Table 8. The least-square means of ME classes and their 95% confidence intervals for TNW are shown in Figure 6. There was a clear ranking, with higher proportions of Modern European ancestry being associated with higher TNW. Pairwise significance testing indicated that medium levels of ME were significantly different from low ME. The findings are similar to a study that compared local versus exotic pigs in Benin and showed the latter weaned more piglets (Dotche et al., 2020b). Further, crossbred pigs weaned around three piglets more than local pigs in a study in India (Nath et al., 2013).

**TABLE 8:** The least square means for TNW for the interaction between ME and geographic location and ME.

ME	Geographic location	LS mean	SE
Low	Hoima	7.39^a^	0.98
	Kamuli	4.10^b^	0.96
Medium	Hoima	7.13^a^	1.20
	Kamuli	6.74^a^	1.24
High	Hoima	10.4^a^	1.55
	Kamuli	5.94^b^	0.54

^a^ LS, means with different superscripts in each ME, category are significantly different.

^b^ LS, means with different superscripts in each ME, category are significantly different.

## 4 Conclusion

Genetic and environmental factors influence phenotypes. In this study, we analyzed the effects of the proportion of Modern European ancestry of smallholder pigs in Uganda on growth and litter size traits. The variation in ancestry levels was limited, with none of the animals having more than 50% Modern European (Large White and Landrace) ancestry. The growth rates of pigs were extremely low, being around 55 g per day for an age range from 7 to 210 days. Further, while ME did not have a significant effect on growth, growth was significantly affected by the housing system as reported in this study. These findings underscore the role of appropriate management interventions for improved growth performance. Sow reproductive performance was influenced by parity for both TBA and TNW. Additionally, ME had a significant effect on TNW, such that sows with high ME ancestry weaned close to three piglets more than sows with low ME ancestry. These findings underscore the role of genetics and appropriate management for improved productivity of pigs in smallholder herds in Uganda.

## Data Availability

The genomic data for samples collected from Uganda and used in this study is publically available here: Dryad https://doi.org/10.5061/dryad.qnk98sfm9.
